# Endothelitis profile in acute heart failure and cardiogenic shock patients: Endocan as a potential novel biomarker and putative therapeutic target

**DOI:** 10.3389/fphys.2022.965611

**Published:** 2022-08-11

**Authors:** Marta Reina-Couto, Carolina Silva-Pereira, Patrícia Pereira-Terra, Janete Quelhas-Santos, João Bessa, Paula Serrão, Joana Afonso, Sandra Martins, Cláudia Camila Dias, Manuela Morato, João T Guimarães, Roberto Roncon-Albuquerque, José-Artur Paiva, António Albino-Teixeira, Teresa Sousa

**Affiliations:** ^1^ Departamento de Biomedicina—Unidade de Farmacologia e Terapêutica, Faculdade de Medicina da Universidade do Porto (FMUP), Porto, Portugal; ^2^ Centro de Investigação Farmacológica e Inovação Medicamentosa, Universidade do Porto (MedInUP), Porto, Portugal; ^3^ Serviço de Medicina Intensiva, Centro Hospitalar Universitário São João (CHUSJ), Porto, Portugal; ^4^ Serviço de Farmacologia Clínica, CHUSJ, Porto, Portugal; ^5^ Serviço de Patologia Clínica, CHUSJ and EPIUnit, Instituto de Saúde Pública, Universidade do Porto, Porto, Portugal; ^6^ Departamento de Medicina da Comunidade, Informação e Decisão em Saúde, FMUP, Porto, Portugal; ^7^ CINTESIS—Centro de Investigação em Tecnologias e Serviços de Saúde, Porto, Portugal; ^8^ Laboratório de Farmacologia, Departamento de Ciências do Medicamento, Faculdade de Farmácia da Universidade do Porto, Porto, Portugal; ^9^ LAQV/REQUIMTE, Faculdade de Farmácia, Universidade do Porto, Porto, Portugal; ^10^ Departamento de Biomedicina—Unidade de Bioquímica, FMUP, Porto, Portugal; ^11^ Departamento de Cirurgia e Fisiologia, FMUP, Porto, Portugal; ^12^ Departamento de Medicina, FMUP, Porto, Portugal

**Keywords:** endocan, endothelitis, acute heart failure, cardiogenic shock, biomarker

## Abstract

**Aims:** Inflammation-driven endothelitis seems to be a hallmark of acute heart failure (AHF) and cardiogenic shock (CS). Endocan, a soluble proteoglycan secreted by the activated endothelium, contributes to inflammation and endothelial dysfunction, but has been scarcely explored in human AHF. We aimed to evaluate serum (S-Endocan) and urinary endocan (U-Endocan) profiles in AHF and CS patients and to correlate them with biomarkers/parameters of inflammation, endothelial activation, cardiovascular dysfunction and prognosis.

**Methods:** Blood and spot urine were collected from patients with AHF (*n* = 23) or CS (*n* = 25) at days 1–2 (admission), 3-4 and 5-8 and from controls (blood donors, *n* = 22) at a single time point. S-Endocan, U-Endocan, serum IL-1β, IL-6, tumour necrosis factor-α (S-TNF-α), intercellular adhesion molecule-1 (S-ICAM-1), vascular cell adhesion molecule-1 (S-VCAM-1) and E-selectin were determined by ELISA or multiplex immunoassays. Serum C-reactive protein (S-CRP), plasma B-type natriuretic peptide (P-BNP) and high-sensitivity troponin I (P-hs-trop I), lactate, urea, creatinine and urinary proteins, as well as prognostic scores (APACHE II, SAPS II) and echocardiographic left ventricular ejection fraction (LVEF) were also evaluated.

**Results:** Admission S-Endocan was higher in both patient groups, with CS presenting greater values than AHF (AHF and CS vs. Controls, *p* < 0.001; CS *vs*. AHF, *p* < 0.01). Admission U-Endocan was only higher in CS patients (*p* < 0.01 *vs*. Controls). At admission, S-VCAM-1, S-IL-6 and S-TNF-α were also higher in both patient groups but there were no differences in S-E-selectin and S-IL-1β among the groups, nor in P-BNP, S-CRP or renal function between AHF and CS. Neither endocan nor other endothelial and inflammatory markers were reduced during hospitalization (*p* > 0.05). S-Endocan positively correlated with S-VCAM-1, S-IL-6, S-CRP, APACHE II and SAPS II scores and was positively associated with P-BNP in multivariate analyses. Admission S-Endocan raised in line with LVEF impairment (*p* = 0.008 for linear trend).

**Conclusion:** Admission endocan significantly increases across AHF spectrum. The lack of reduction in endothelial and inflammatory markers throughout hospitalization suggests a perpetuation of endothelial dysfunction and inflammation. S-Endocan appears to be a biomarker of endothelitis and a putative therapeutic target in AHF and CS, given its association with LVEF impairment and P-BNP and its positive correlation with prognostic scores.

## Introduction

Acute heart failure (AHF) is broadly defined as a rapid onset of new or worsening signs and symptoms of heart failure (HF) (McDonagh et al., 2021). These are due to an increase in left ventricular (LV) filling pressure with consequent pulmonary congestion but not necessarily associated with low cardiac output - both HF with reduced or preserved ejection fraction seem to have similar hospital admissions and readmissions (Metra and Teerlink, 2017). In fact, the economic burden of HF is becoming one of the most problematic public health issues with increasing prevalence and costs. Due to the ageing of the population and progressive treatment of HF with significant reduction of mortality (Benjamin et al., 2019; Groenewegen et al., 2020), it represents nowadays the most common diagnosis in patients over 65 years-old with unscheduled admission to hospital in high-income nations (Braunwald, 2015). It also reflects our poor pathophysiologic knowledge because no AHF clinical trials have shown to improve in-hospital symptoms and postdischarge clinical outcomes compared with placebo (Tomasoni et al., 2019), except maybe recently for sacubitril/valsartan where early titration before discharge was demonstrated to be associated with favourable reduction of natriuretic biomarkers, an accepted surrogate for prognostication (Velazquez et al., 2019; Wachter et al., 2019).

From what is known so far, pathophysiology in AHF is presently viewed as consisting theoretically of two phases - initiation and amplification phase (Sabbah, 2017) - in a spiral of multiple concurrent mechanisms contributing for worsening HF and end-organ damage (Harjola et al., 2017). A progressive chronic energetic exhaustion of the failing myocardium or a crescendo of vascular stiffness dependent on neurohormonal activation (Mentz and O'Connor, 2016) with inflammation (Reina-Couto et al., 2021) and endothelial disruption, fluid accumulation and cardiac vicious workload (Colombo et al., 2008) results in the clinical cardinal sign of congestion in most patients with AHF, but a smaller proportion presents with peripheral hypoperfusion or cardiogenic shock (CS) (Chioncel et al., 2017b). This most severe form of AHF remains with an unchanged mortality as high as 50% and also with few evidence-based effective therapeutics (Thiele et al., 2019). Interestingly, we still cannot conclude whether congestion is cause or consequence of endothelial dysfunction (Colombo et al., 2014; Colombo et al., 2015) which remains to be explored in AHF. Nevertheless, even though endothelial dysfunction prognostic significance is well recognized in chronic HF (Alem, 2019; Zuchi et al., 2020), the classical methods for its evaluation are technically challenging and have limited its clinical implementation (Shantsila et al., 2012). Also, the assessment of systemic endothelial biomarkers is not yet consolidated for clinical practice, namely for AHF.

Endocan, previously designated as endothelial cell-specific molecule-1 (ESM-1), is a soluble dermatan sulfate proteoglycan synthetized specifically by endothelial cells, probably mirroring inflammation-driven “endothelitis” (Lassalle et al., 1996). There has been an increasing interest in exploring endocan’s utility as a biomarker in a wide spectrum of pathological states, particularly in sepsis, acute respiratory distress syndrome and several cardiovascular diseases (De Freitas Caires et al., 2018; Bessa et al., 2020). Increased values independently related with prognosis and cardiovascular events have been recently reported for endocan in coronary and chronic HF patients (Kosir et al., 2019; Ziaee et al., 2019) and its therapeutic potential has just started to be explored for cancer (Zhang et al., 2021). As it can be easily detected in the human bloodstream and was proved to be stable, intact and reliable in critical patients (Gaudet et al., 2017), we aimed to explore serum and urinary endocan profiles in AHF and CS patients, where endothelial activation and congestion seem to be plausible and specific hallmarks. Furthermore, we analysed their correlation with prognostic parameters as well as with mechanistic biomarkers of inflammation, endothelial activation or cardiovascular dysfunction.

## Materials and methods

### Study design and population

The present study is part of a larger research project (RIFF-HEART—Resolution of inflammation: a missing key to improve acute heart failure treatment and prognosis?) involving patients admitted to the Service of Intensive Care Medicine of a tertiary hospital (Centro Hospitalar Universitário São João, CHUSJ).

We performed a single-centre cohort study and consecutively recruited patients with a single diagnosis of AHF (*n* = 23) or CS (*n* = 25) admitted to the Service of Intensive Care Medicine of CHUSJ, from January 2017 to December 2019. Controls (*n* = 22) were recruited among healthy blood donor volunteers from the Service of Immunohemotherapy of CHUSJ, from September 2017 to October 2017. All eligible patients (or their legal representative) provided written informed consent to participate in the study. Blood donor volunteers provided verbal informed consent. The study protocol conforms to the Guidelines for Good Clinical Practice and the ethical guidelines of the 1975 Declaration of Helsinki. The study was approved by the institution’s ethics committee (CES 75-16).

### Clinical data and sample collection

Physical examination of the patients was performed during their Intensive Care Unit (ICU) stay and a record of demographic and clinical data was completed by the medical team of the project and anonymously coded to the project database, along with laboratory data, guaranteeing confidentiality. Illness severity was assessed by the Acute Physiology and Chronic Health Evaluation II (APACHE II) and Simplified Acute Physiology Score II (SAPS II) scoring systems at ICU admission, as well as by the values of the natriuretic peptide in use in our centre (plasma B-type natriuretic peptide, P-BNP) and the echocardiographic LV ejection fraction (LVEF). ICU length of stay, total hospital length of stay, in-hospital mortality and mortality at 1 year were also evaluated.

Blood and spot urine samples were collected in patients (AHF and CS groups) at 3 different time points during ICU stay, whenever possible: up to 48 h (days 1–2, admission), on days 3–4 and on days 5–8 after ICU admission. All samples from CS patients on mechanical circulatory support were obtained at days 1–2, days 3–4 and days 5–8 after veno-arterial extracorporeal membrane oxygenation (VA-ECMO) initiation which coincided with ICU admission. Samples (blood and spot urine) from controls were collected at a single time point. All samples were processed within 1–2 h of collection and stored at −80°C until assayed.

### Routine clinical biochemical and cardiac markers

P-BNP and plasma high-sensitivity troponin I (P-hs-trop I) were measured by chemiluminescent microparticle immunoassays using an Abbot^®^ Architect i2000 automated analyser (Abbott^®^ Diagnostics, Lake Forest, IL, USA). A Beckman Coulter^®^ AU5400 automated clinical chemistry analyser (Beckman Coulter^®^, Portugal) was used for the quantification of serum C-reactive protein (S-CRP) by an immunoturbidimetric assay, serum urea (S-Urea) concentration by a kinetic urease/glutamate dehydrogenase method, plasma and urine creatinine by the colorimetric Jaffe method and urinary protein by the pyrogallol red method. Lactate was evaluated by blood gas analysis.

### Quantification of serum and urinary endocan

Serum and urinary endocan (S-Endocan and U-Endocan, respectively) were quantified by enzyme-linked immunosorbent assays (ELISA) using commercial kits (S-Endocan: “Just Do It ELISA Kit H1”, JDIEK H1, Lunginnov, Lille, France; U-Endocan: Human ESM1/Endocan ELISA Kit, LS-F24487, LSBio, Inc., Seattle, USA). U-Endocan values were corrected for urinary creatinine concentrations.

### Quantification of other biomarkers of endothelial activation and inflammation

Other serum endothelium activation markers (serum intercellular adhesion molecule 1, S-ICAM-1; serum vascular cell adhesion molecule 1, S-VCAM-1; serum E-Selectin, S-E-Selectin) and proinflammatory cytokines (serum interleukin 6, S-IL-6; serum interleukin one beta, S-IL-1β; serum tumour necrosis factor alpha, S-TNF-α) were evaluated by multiplex immunoassays using a Luminex 200™ xMAP™ analyzer (Luminex Corporation, Austin, TX, USA), according to the protocols of Human Premixed Multi-Analyte Magnetic Assay (R&D Systems, Inc., Minneapolis, USA) and Human High Sensitivity T Cell Magnetic Bead Panel (Milliplex^®^ Map kit, Millipore Corporation, Billerica, MA, USA), respectively. Raw data analysis (mean fluorescence intensity) was performed using a standard five parameter logistic (5-PL) curve fit created by the Luminex xPONENT ^®^ Software (version 3.1).

### Data and statistical analysis

**FIGURE 1:**
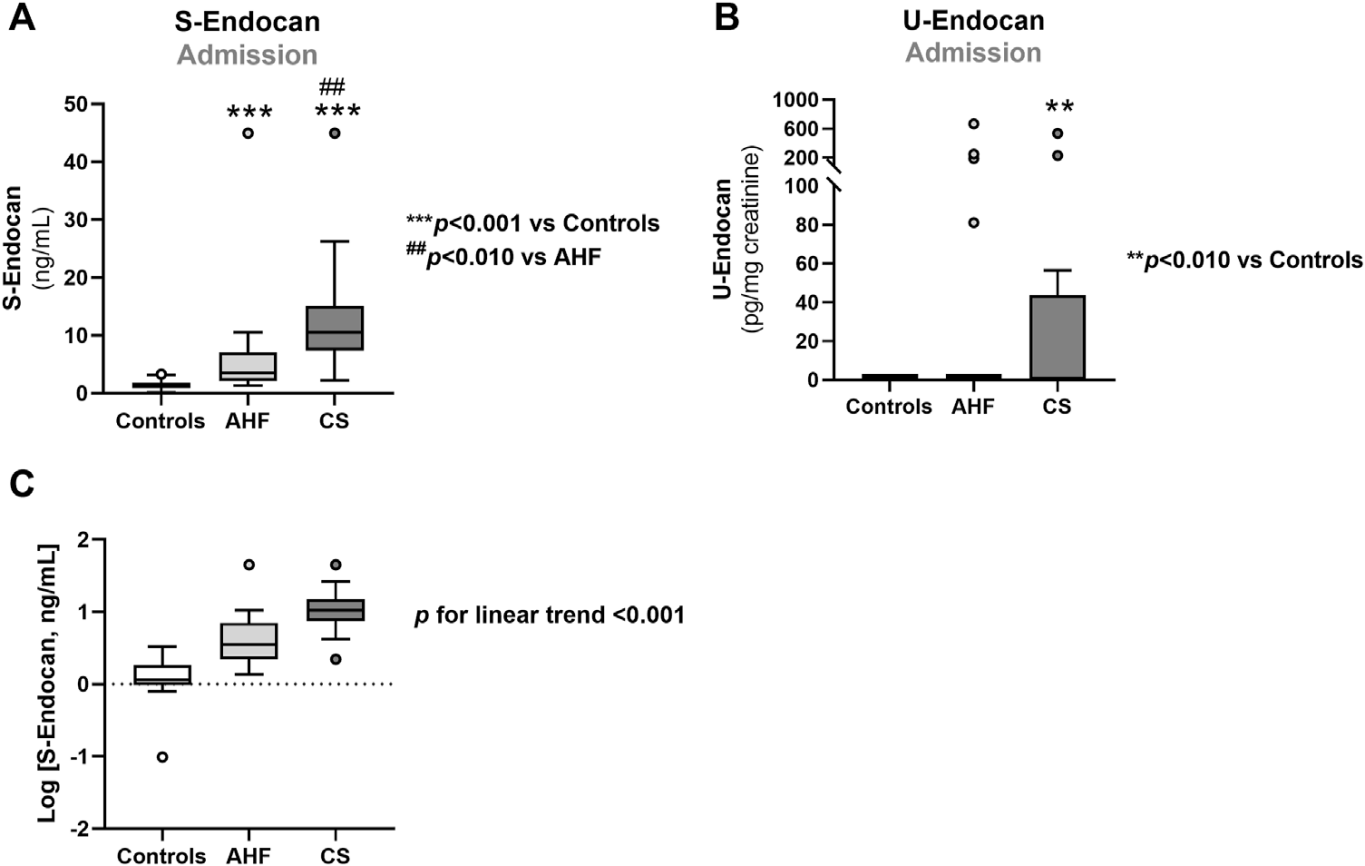
Serum endocan **(A,C)** and urinary endocan **(B)** concentrations at admission in controls, CS and AHF patients. Results are presented in Box-and-Whiskers plot. AHF, acute heart failure; CS, cardiogenic shock; S-Endocan, serum endocan; U-Endocan, urinary endocan.

**FIGURE 2:**
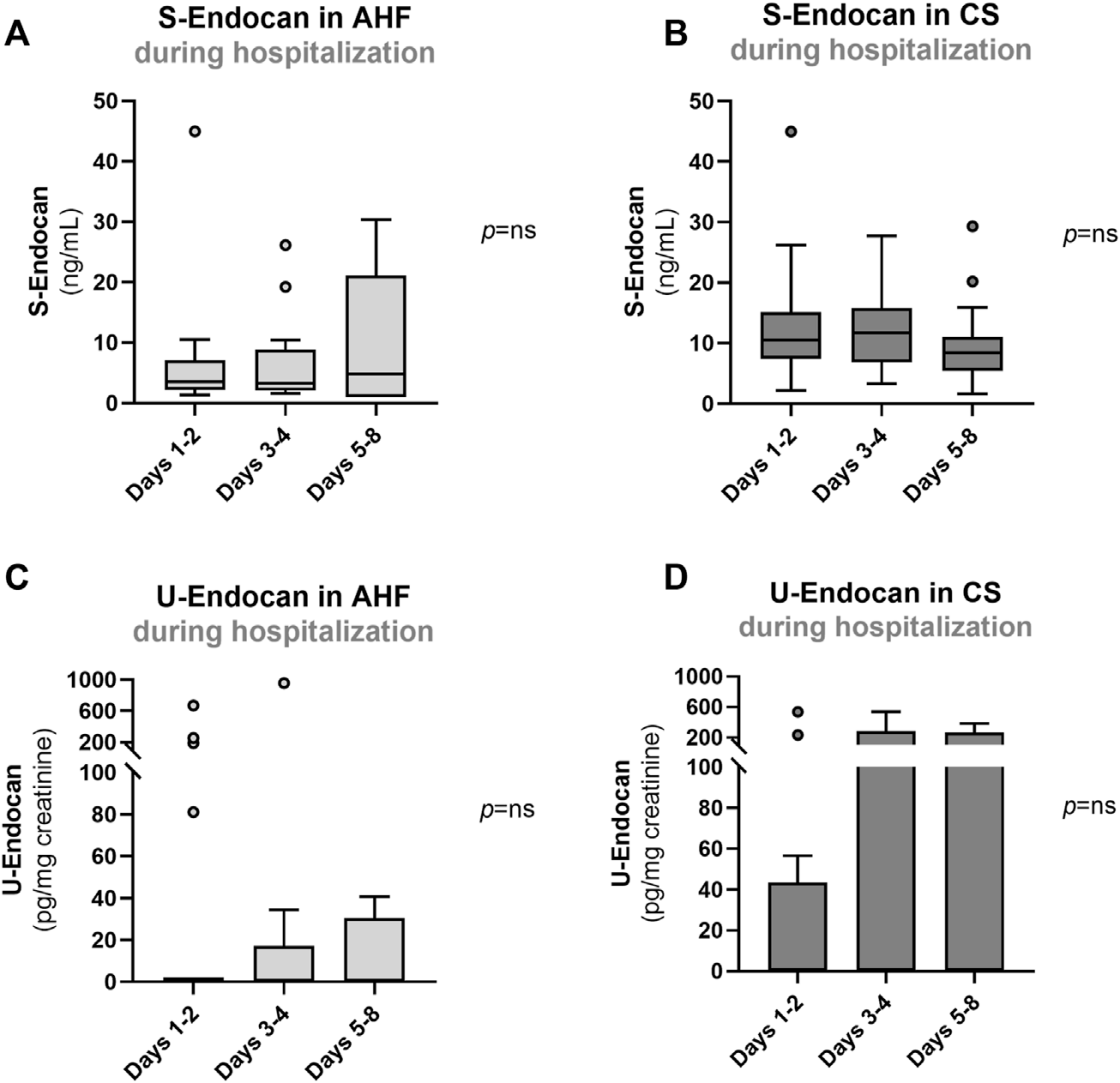
Serum **(A, B)** and urinary endocan **(C, D)** at days 1–2, days 3–4 and 5–8 in patients with AHF or with CS. Results are presented in Box-and-Whiskers plot. AHF, acute heart failure; CS, cardiogenic shock; S-Endocan, serum endocan; U-Endocan, urinary endocan.

**FIGURE 3:**
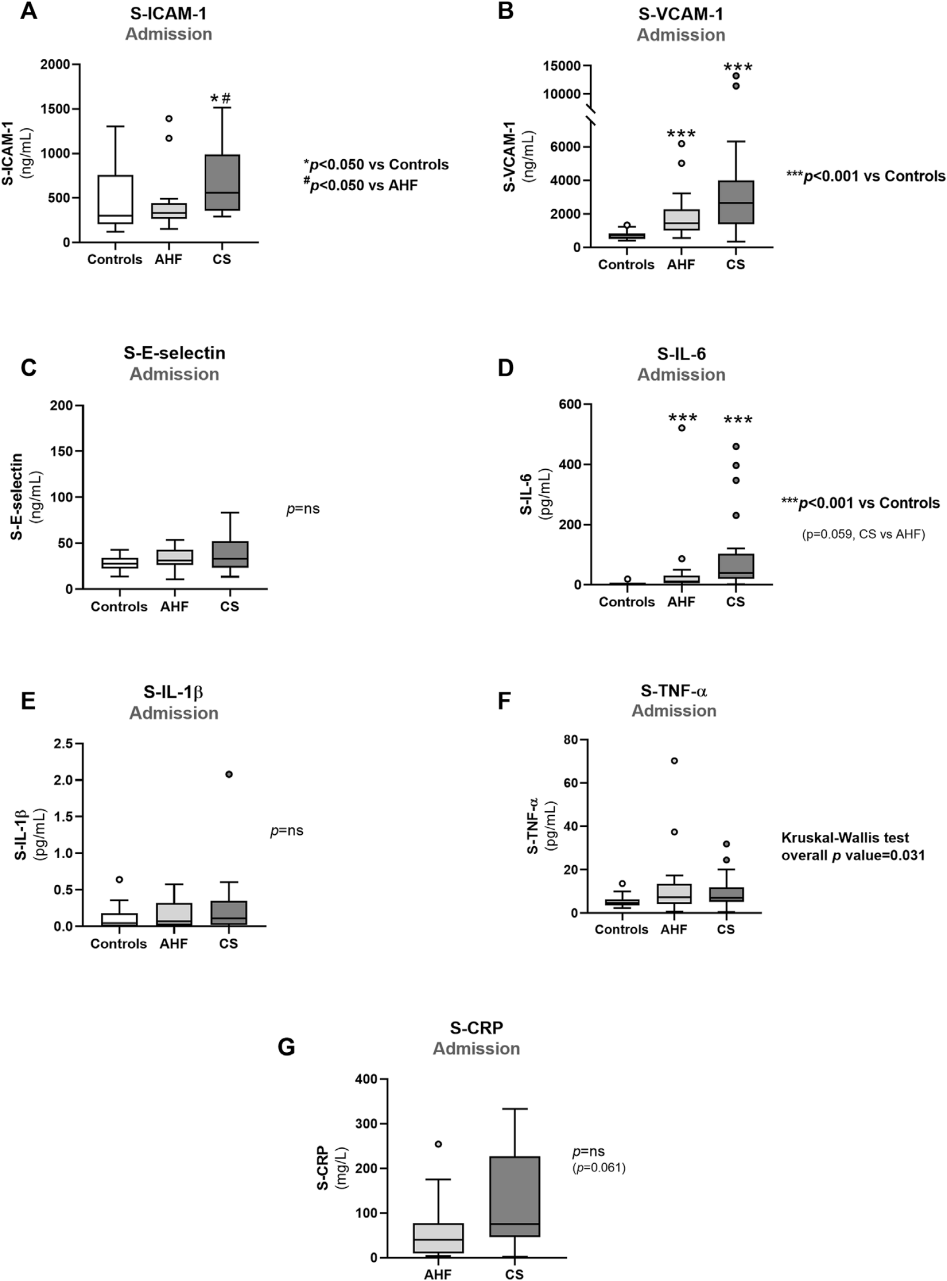
Admission values of biomarkers of endothelial dysfunction and inflammation in controls, AHF and CS: **(A)** S-ICAM-1; **(B)** S-VCAM-1); **(C)** S-E-Selectin; **(D)** S-IL-6; **(E)** S-IL-1β; **(F)** S-TNF-α; **(G)** S-CRP. Results are presented in Box-and-Whiskers plot. AHF, acute heart failure; CS, cardiogenic shock; S-CRP, serum C-reactive protein; S-E-Selectin, serum E-Selectin; S-ICAM-1, serum intercellular adhesion molecule 1; S-IL-1β, serum interleukin 1β; S-IL-6, serum interleukin 6; S-TNF-α, serum tumour necrosis factor alpha; S-VCAM-1, serum vascular cell adhesion molecule 1.

**FIGURE 4:**
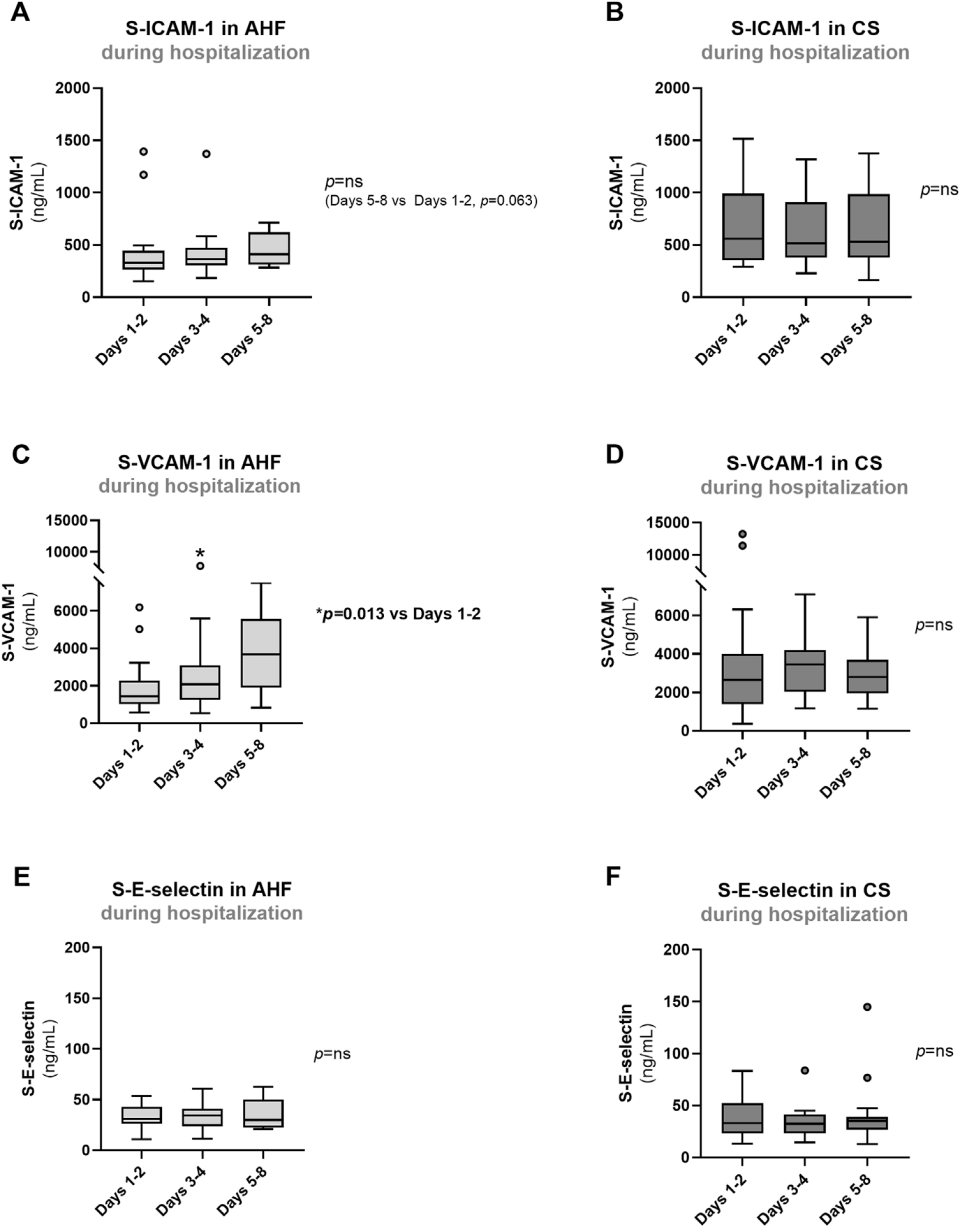
Evolution of biomarkers of endothelial dysfunction in AHF and CS patients during hospitalization: **(A,B)** S-ICAM-1; **(C,D)** S-VCAM-1); **(E,F)** S-E-Selectin. Results are presented in Box-and-Whiskers plot. AHF, acute heart failure; CS, cardiogenic shock; S-E-Selectin, serum E-Selectin; S-ICAM-1, serum intercellular adhesion molecule 1; S-VCAM-1, serum vascular cell adhesion molecule 1.

**FIGURE 5:**
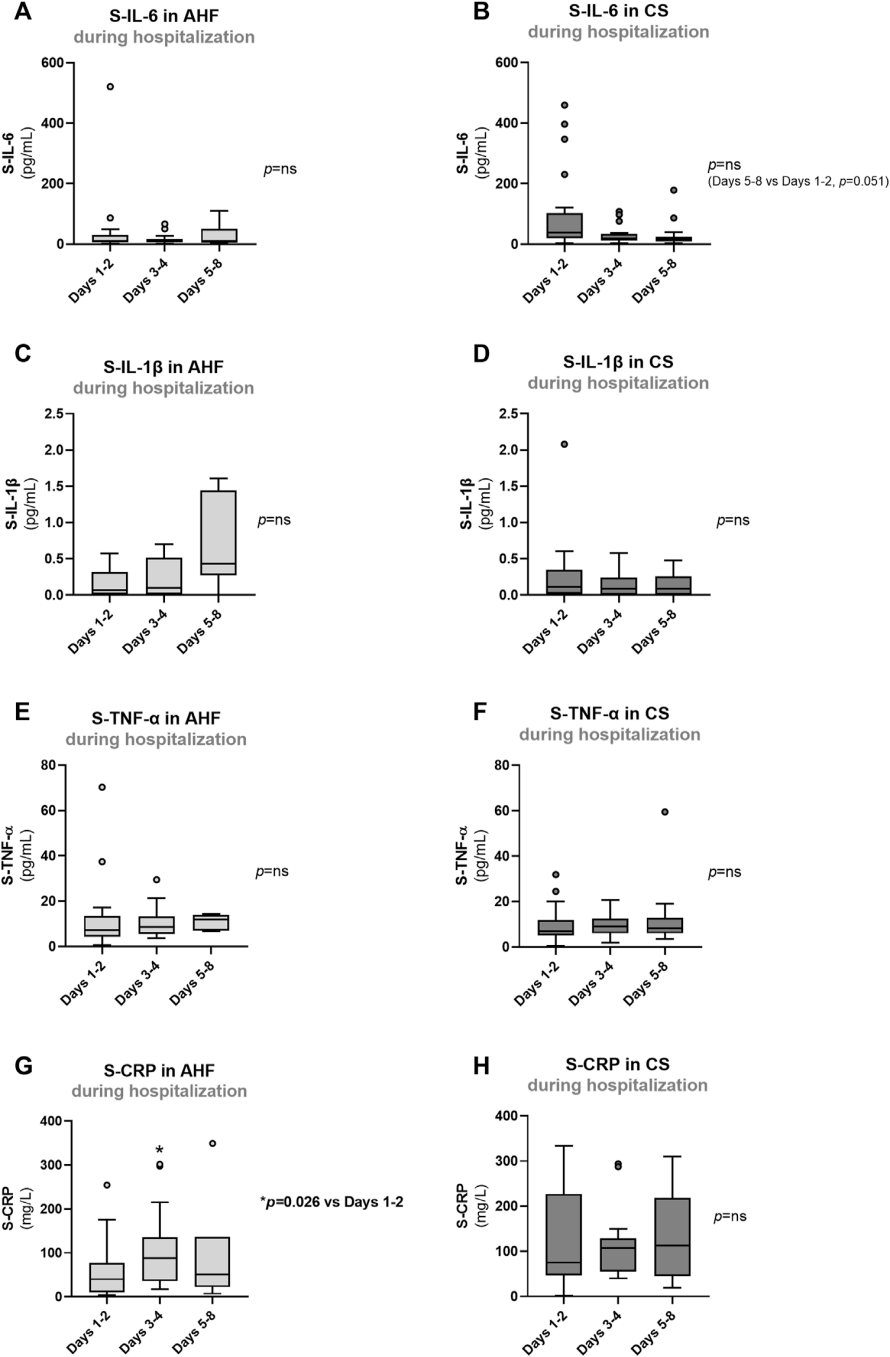
Evolution of biomarkers of inflammation in AHF and CS patients during hospitalization: **(A,B)** S-IL-6; **(C,D)** S-IL-1β; **(E,F)** S-TNF-α; **(G,H)** S-CRP. Results are presented in Box-and-Whiskers plot. AHF, acute heart failure; CS, cardiogenic shock; S-CRP, serum C-reactive protein; S-IL-1β, serum interleukin 1β; S-IL-6, serum interleukin 6; S-TNF-α, serum tumour necrosis factor alpha.

**FIGURE 6:**
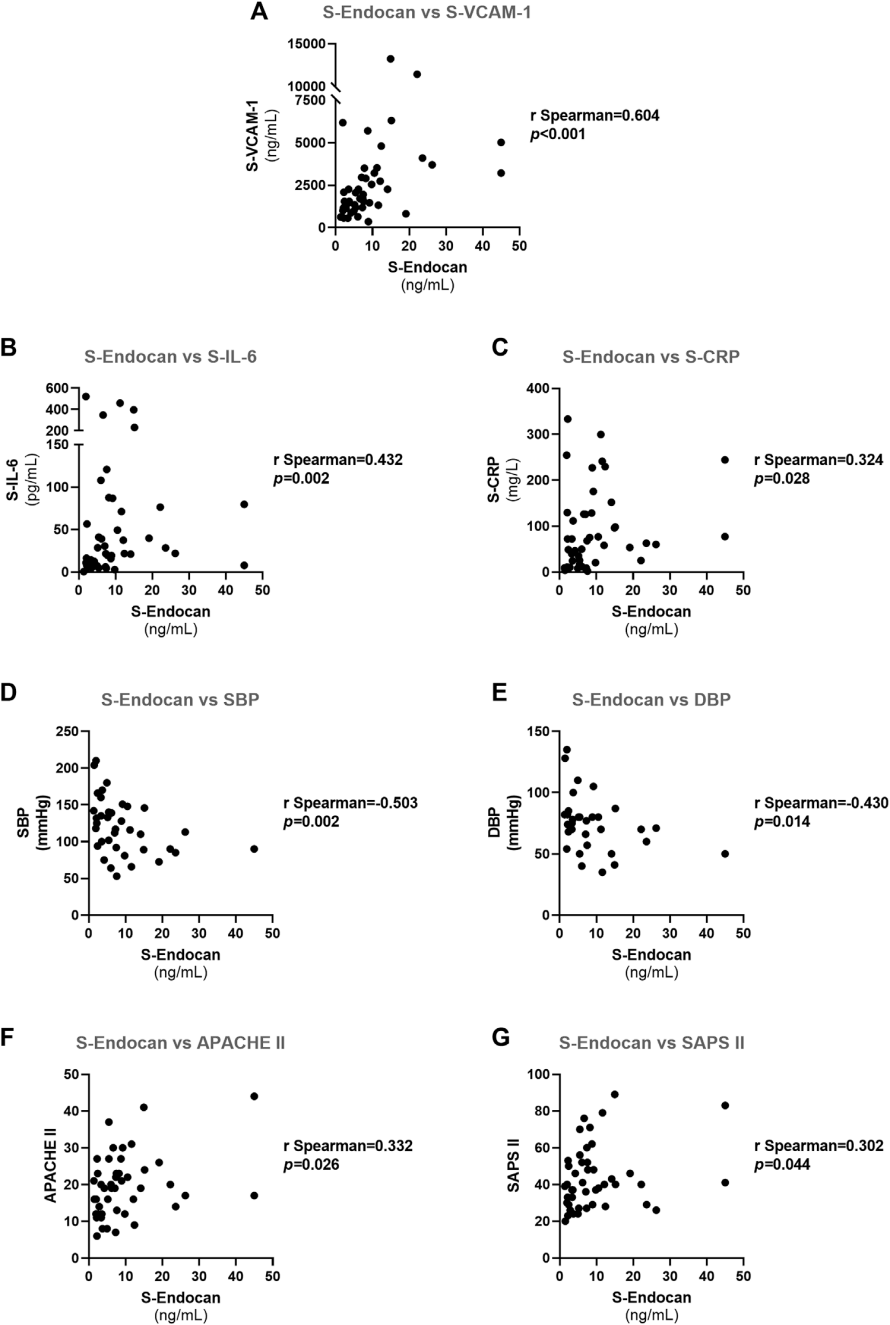
Spearman correlations for serum endocan in all patients at admission: **(A)** S-Endocan *vs*. S-VCAM-1; **(B)** S-Endocan *vs*. S-IL-6; **(C)** S-Endocan *vs*. S-CRP; **(D)** S-Endocan *vs*. S-SBP; **(E)** S-Endocan *vs*. DBP; **(F)** S-Endocan *vs*. APACHE II; **(G)** S-Endocan *vs*. SAPS II. APACHE II, acute physiology and chronic health evaluation II; DBP, diastolic blood pressure; SAPS II, simplified acute physiology score (SAPS) II; SBP, systolic blood pressure; S-Endocan, serum endocan; S-CRP, serum C-reactive protein; S-IL-6, serum interleukin 6; S-VCAM-1, serum vascular cell adhesion molecule 1.

**FIGURE 7:**
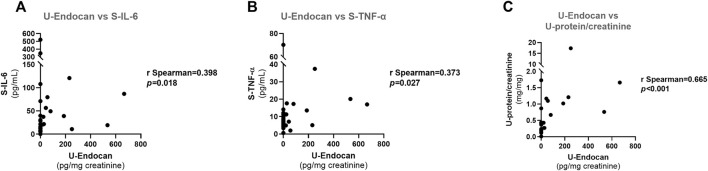
Spearman correlations for urinary endocan in all patients at admission: **(A)** U-Endocan *vs*. S-IL-6; **(B)** U-Endocan *vs*. S-TNF-α; **(C)** U-Endocan *vs*. U-protein/creatinine. S-IL-6, serum interleukin 6; S-TNF-α, serum tumour necrosis factor alpha; U-Endocan, urinary endocan; U-protein/creatinine, urinary protein/creatinine.

**FIGURE 8:**
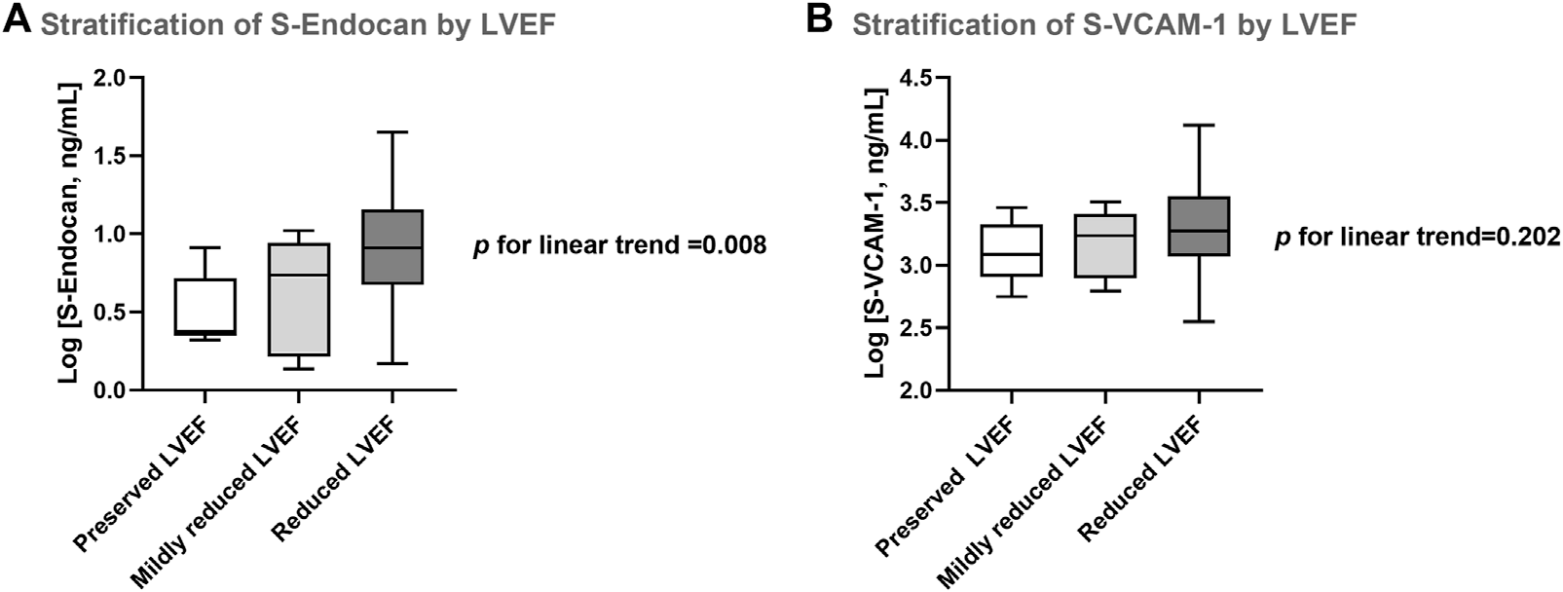
Serum endocan **(A)** and VCAM-1 **(B)** stratified by left ventricular ejection fraction (LVEF). Preserved LVEF: LVEF ≥50%; mildly reduced LVEF: LVEF 41–49%; reduced LVEF: LVEF ≤40%. Results are presented in Box-and-Whiskers plot. S-Endocan, serum endocan; S-VCAM-1, serum vascular cell adhesion molecule 1.

Results are expressed as mean ± standard error of the mean (SEM) or as median (25th percentile; 75th percentile) for data with normal or non-normal distribution, respectively, or as percentage, and are graphically represented as Box and Whiskers plots (Figures 1–5 and Figure 8) or as scatter plots (Figures 6, 7) or as Kaplan-Meier plot (survival analysis; Supplementary Figure S1). Estimated glomerular filtration rate (eGFR) was calculated using the Chronic Kidney Disease Epidemiology Collaboration (CKD-EPI) equation (Levey et al., 2009). Statistical analysis was conducted using the GraphPad Prism 9 software (La Jolla, USA) and the IBM SPSS Statistics 27 software (IBM Corporation, New York, USA). Results were analysed by unpaired Student’s t-test or Mann–Whitney *U*-test, for comparisons between two groups, or by one-way ANOVA followed by a Tukey’s multiple comparison test or a Kruskal–Wallis test followed by a Dunn’s post hoc test, for comparison between three groups, where appropriate. The value of *p* for linear trend was estimated by one-way ANOVA followed by a post hoc test for trend, after applying a base 10 log transformation to the variables with non-normal distribution. Categorical variables were analysed by the Fisher’s exact test or by the Chi-Square test. Biomarkers evolution throughout the hospitalization was analysed by Wilcoxon matched pairs signed rank test. Spearman’s correlation analysis was used to estimate correlations between sets of nonparametric data in AHF and CS patients at admission. All *p* values of <0.050 were considered significant.

Repeated measures multivariate analyses were conducted to determine the relationship between S-Endocan (as dependent variable) and endothelial activation markers (S-VCAM-1), inflammation (S-IL-6), cardiac markers (P-BNP and P-hs-trop I), systolic and diastolic blood pressure (SBP and DBP, respectively) and prognostic scores, adjusted for some confounders, namely age and gender.

The ability of S-Endocan, U-Endocan, S-VCAM-1, P-BNP and APACHE II and SAPS II scores to discriminate in-hospital mortality was evaluated by plotting receiver operating characteristic (ROC) curves and computing the area under the curve (AUC).

To prevent possible bias in clinical evaluation, all the patients were examined by the same medical team included in the project. To assure comparability of biomarkers assessment, samples from controls, AHF and CS groups were evenly distributed in each assay plate. There were missing values in some biomarkers due to insufficient volume to perform sample processing, dilution tests and assays. In addition, some routine clinical biomarkers could only be assessed at a single time point during hospitalization due to hospital’s internal policies, and we had not permission to measure routine clinical biomarkers in controls (blood donor volunteers), except for creatinine, or to have access to their hospital laboratory reports. The final number per group for the biomarkers/parameters evaluated at admission was as following: APACHE II score, *n* = 23 *vs*. 23 (AHF *vs*. CS); P-BNP, *n* = 20 *vs*. 17 (AHF *vs*. CS); P-hs-trop I, *n* = 23 *vs*. 23 (AHF *vs*. CS); eGFR, *n* = 22 *vs*. 23 *vs*. 23 (Controls *vs.* AHF *vs*. CS); SAPS II score, *n* = 23 *vs*. 23 (AHF vs. CS); S-Urea, *n* = 23 *vs*. 23 (AHF vs. CS); U-protein/creatinine ratio, *n* = 22 *vs*. 15 (AHF vs. CS); S-ICAM-1, *n* = 22 *vs*. 23 *vs.* 24 (Controls vs. AHF *vs*. CS); S-VCAM-1, *n* = 22 *vs*. 23 *vs*. 24 (Controls *vs*. AHF *vs*. CS); S-E-Selectin, *n* = 22 *vs*. 23 *vs*. 24 (Controls *vs*. AHF *vs*. CS); S-TNF-α, n = 22 *vs*. 23 *vs*. 24 (Controls *vs*. AHF *vs*. CS); S-IL-1β, *n* = 22 *vs*. 23 *vs*. 24 (Controls *vs*. AHF vs. CS); S-IL-6, *n* = 22 *vs*. 23 *vs*. 24 (Controls *vs*. AHF *vs*. CS); S-CRP, *n* = 23 *vs*. 23 (AHF *vs*. CS). To avoid biasing the results, no imputation for missing values was used.

Preliminary values of S-Endocan (putative marker for endothelitis) obtained in controls and in patients with AHF or CS were used to estimate sample size by G Power 3.1 software. We found that a sample size of 6 participants/patients in each group would be sufficient to obtain a statistical study power of 80% at a 5% level of significance. The final sample size was defined according to the RIFF-HEART project’s primary objectives that consisted in characterizing not only endothelitis, but also resolution of inflammation markers at admission and during hospitalization.

Reporting of the study conforms to STROBE statement along with references to STROBE and the broader EQUATOR guidelines (Simera et al., 2010).

## Results

### Population demographic, clinical and biochemical characterization

General demographic characteristics, clinical and biochemical parameters are shown in Table 1.

**TABLE 1:** Demographic, clinical and biochemical characteristics at admission and outcomes of study population.

Demographic, clinical and biochemical parameters	Controls (*n* = 22)	AHF (*n* = 23)	CS (*n* = 25)	*p* value
**Age (Years)**	56 ± 1	70 ± 3**	58 ± 4^##^	**<0.001**
Gender, n (%)	—	—	—	0.064^a^
Men	13 (59)	11 (48)	20 (80)	—
Women	9 (41)	12 (52)	5 (20)	—
**APACHE II Score**	n/a	16 (11; 21)	21 (16; 27)	**0.010**
**SAPS II Score**	n/a	36 (27; 41)	46 (37; 62)	**0.004**
**VA-ECMO, n (%)**	n/a	0 (0)	12 (48)	**<0.001** ^b^
Lactate (mmol/L)	n/a	1.28 (1.05; 1.70)	1.47 (1.17; 2.75)	0.092
Precipitating factors on admission, n (%)				
Acute coronary syndrome	n/a	9 (39)	10 (40)	>0.999^b^
Arrhythmia	n/a	3 (13)	4 (16)	>0.999^b^
ADHF	n/a	5 (22)	3 (12)	0.366^b^
Valvular disease	n/a	2 (9)	1 (4)	0.502^b^
Infection	n/a	1 (4)	5 (20)	0.101^b^
Other	n/a	3 (13)	1 (4)	0.257^b^
Unknown	n/a	0 (0)	1 (4)	0.332^b^
Comorbidities, n (%)				
**Arterial Hypertension**	n.d	18 (78)	12 (48)	**0.031** ^b^
Diabetes	n.d	13 (57)	8 (32)	0.087^b^
Obesity	n.d	3 (13)	1 (4)	0.257^b^
**Dyslipidemia**	n.d	13 (57)	4 (16)	**0.005** ^b^
Chronic pulmonary disease	n.d	3 (13)	2 (8)	0.568^b^
Chronic kidney disease	n.d	4 (17)	1 (4)	0.129^b^
Cancer	n.d	1 (4)	0 (0)	0.292^b^
Anemia	n.d	12 (52)	17 (68)	0.263^b^
Cardiac biomarkers				
P-BNP (pg/ml)	n.d	897 (417; 1542)	869 (321; 2691)	>0.999
**P-hs-trop I (ng/L)**	n.d	423 (114; 11,586)	9311 (209; 105,413)	**0.030**
Renal function				
eGFR (mL/min per 1.73 m^2^)	77 ± 4	71 ± 6	70 ± 5	0.566
S-Urea (mg/dl)	n.d	54 (37; 77)	66 (44; 73)	0.436
U-protein/creatinine (mg/mg)	n.d	0.3 (0.1; 0.7)	0.4 (0.2; 1.1)	0.458
Previous therapeutics, n (%)				
**RAAS inhibitors (ACE inhibitor and/or ARB)**	n.d	17 (74)	10 (40)	**0.029** ^b^
Therapeutics during hospitalization, n (%)
Statins	n.d	18 (78)	13 (52)	0.075^b^
Aspirin	n.d	14 (61)	14 (56)	0.777^b^
P2Y_12_ receptor antagonists	n.d	10 (43)	10 (40)	>0.999^b^
Outcomes				
**ICU length of stay (days)**	n/a	4 (3; 7)	10 (5; 21)	**0.002**
Total hospital length of stay (days)	n/a	12 (9; 18)	14 (6; 22)	0.716
In-hospital mortality, n (%)	n/a	5 (22)	11 (44)	0.132^b^
Mortality within 12 months, n (%)	n/a	8 (35)	16 (64)	0.082^b^

ACE, angiotensin converting enzyme; ADHF, acute decompensated heart failure; AHF, acute heart failure; APACHE II, Acute Physiology And Chronic Health Evaluation II; ARB, angiotensin II, receptor blocker; CS, cardiogenic shock; eGFR, estimated glomerular filtration rate; ICU, intensive care unit; n/a, not applicable; n.d., not determined; P-BNP, plasma B-type natriuretic peptide; P-hs-trop I, plasma high-sensitivity troponin I; RAAS, renin-angiotensin-aldosterone system; SAPS II, Simplified Acute Physiology Score II; S-urea; serum urea; U-protein/creatinine, urinary protein/creatinine; VA-ECMO, venoarterial extracorporeal membrane oxygenation. Results are expressed as number (%), mean ± SEM, or as median (25th percentile; 75th percentile) for data with normal or non-normal distribution, respectively. **p < 0.01 *vs*. Controls; ^##^ *p* < 0.01 *vs*. AHF. All the parameters that have significant *p* values (*p* < 0.05) should be written in bold (both the parameter and the respective *p* value).

a Chi-Square test

b Fisher’s exact test

In this study, 22 healthy controls and 48 patients, 23 with AHF and 25 with CS, were assessed. Male patients were more prevalent in the CS group (*n* = 20), compared to AHF (*n* = 11) and controls (*n* = 13), with this difference among groups being borderline significant (*p* = 0.064). Patients in the AHF group were significantly older than controls and CS patients (Controls: 56 ± 1 year; AHF: 70 ± 3 years; CS: 58 ± 4 years, *p* < 0.001). As expected, APACHE II and SAPS II scores were significantly higher in the CS group, in which 48% of the patients were supported with VA-ECMO.

Precipitating factors on admission were identical for AHF and CS groups, with acute coronary syndrome being the predominant trigger in both groups, followed by acutely decompensated HF in AHF and infection in CS patients, respectively. Arterial hypertension, diabetes, dyslipidemia and anemia were the most prevalent comorbidities in both groups, but the AHF group presented a significantly higher number of patients with arterial hypertension and dyslipidemia. The AHF group also included a significantly higher number of patients that were being treated with renin-angiotensin-aldosterone system (RAAS) inhibitors prior to admission when compared to the CS group. Regarding therapeutics throughout hospitalization, AHF and CS groups showed no significant differences in the number of patients on statin, aspirin or P2Y_12_ receptor antagonist therapies.

Patients with CS had significantly higher concentration of P-hs-trop I at admission, but no differences were found in P-BNP nor in lactate values when compared to AHF patients. We did not find significant differences in eGFR between all groups or in serum urea and proteinuria at admission between AHF and CS groups. CS patients had a longer length of stay in the ICU than patients with AHF, but there were no differences between these groups regarding the total length of hospital stay. There was a high in-hospital mortality and 1-year mortality in both groups (Table 1). Although no significant differences were observed in mortality parameters between AHF and CS patients, the CS group had a tendentially higher 1-year mortality (CS = 64% *vs*. AHF = 35%, *p* = 0.082) (Table 1). A Kaplan-Meier survival plot is shown in Supplementary Material, Supplementary Figure S1. The median survival time for the ICU patients evaluated was 13 months.

### S-Endocan and U-Endocan at admission and during hospitalization

At admission, S-Endocan was significantly higher in AHF (*p* < 0.001 *vs*. controls) and even higher in CS (*p* < 0.001 *vs*. controls; *p* < 0.010 *vs*. AHF; *p* < 0.001 for linear trend) (Figures 1A,C), whereas U-Endocan was only significantly higher in CS patients (Figure 1B). During hospitalization, we found no significant reduction in S-Endocan (Figures 2A,B) or U-endocan values (Figures 2C,D) in both patient groups.

CS patients that were on RAAS inhibitors prior to admission had significantly lower concentrations of S-Endocan [8.1 (5.1; 12.2) *vs*. 12.4 (8.5; 22.9) ng/ml, *p* = 0.026, Supplementary Table S1] at admission, but no changes were detected in other time points or in U-Endocan or in AHF patients. Treatment with statin, aspirin or P2Y_12_ receptor antagonists throughout hospitalization did not affect S-Endocan or U-Endocan in AHF or CS patients (Supplementary Table S1).

### Other biomarkers of endothelial activation and inflammation

Concerning other endothelial activation biomarkers, we found significantly higher admission values of S-ICAM-1 in CS patients compared to AHF patients and controls (*p* < 0.050). However, admission S-ICAM-1 concentration in AHF patients did not differ from control values (*p* > 0.050) (Figure 3A). Admission S-VCAM-1 was increased by 2-fold in AHF and almost by 4-fold in CS patients compared to controls (*p* < 0.001 for AHF and CS *vs*. controls), but no differences were found in admission S-E-Selectin values among the groups (Figures 3B,C, respectively). Both S-ICAM-1 and S-VCAM-1 concentrations linearly increased across the AHF spectrum (*p* for linear trend = 0.010 for S-ICAM and *p* for linear trend<0.001 for S-VCAM-1).

Patients with AHF or CS exhibited higher admission values of inflammatory markers such as S-IL-6 (*p* < 0.001) and S-TNF-α (overall *p* value = 0.031) compared to controls (Figures 3D,F). There were no significant differences in admission values of S-IL-1β among the groups (Figure 3E) or in admission S-CRP concentrations between AHF and CS groups (Figure 3G). During hospitalization, there was no significant reduction in any of the endothelial and inflammatory markers evaluated (Figures 4, 5). In fact, at days 3–4, we observed a significant increase of S-VCAM-1 and S-CRP in AHF patients (Figures 4C, 5G, respectively).

Treatment with RAAS inhibitors prior to admission was associated with tendentially lower concentrations of S-VCAM-1 [1717 (1108; 2332) *vs*. 3524 (2141; 5252) ng/ml, *p* = 0.058, Supplementary Table S1] in CS patients at admission, but no significant changes were detected in other time points or in other endothelial cell adhesion molecules or in AHF patients (Supplementary Table S1). Statin treatment throughout hospitalization did not affect endothelial cell adhesion molecules in CS patients, but in AHF patients was associated with significantly higher values of E-Selectin at admission and at days 3–4 (Supplementary Table S1). Regarding antiplatelet therapy throughout hospitalization, both aspirin and P2Y_12_ receptor antagonists were associated with opposite effects on endothelial cell adhesion molecules, being associated with a significant reduction of S-VCAM-1 at admission in CS patients and with an increase of S-ICAM-1 at admission and days 3–4 in AHF patients (Supplementary Table S1).

### Comparisons in CS patients with or without VA-ECMO

At admission, there were no significant differences in demographic or in routine biochemical parameters between the VA-ECMO group (*n* = 12) and the conventional medical therapy (CMT) group (*n* = 13), except for the age, the VA-ECMO patients being younger (48 ± 4 years *vs*. 68 ± 4 years, *p* = 0.004, VA-ECMO vs. CMT). During hospitalization, no significant differences were observed between CMT and VA-ECMO groups in S-Endocan, U-Endocan or any of the other endothelial and inflammatory markers evaluated (data not shown), although S-CRP was tendentially higher in the VA-ECMO group at days 5–8 (VA-ECMO: 158.5 (85.6; 223.5 mg/L *vs*. CMT: 55.7 (24.3; 148.4) mg/L, *p* = 0.068).

### Correlation analysis

Within all patients at admission, S-Endocan was inversely correlated with SBP (*r* = −0.503, *p* = 0.002) and DBP (*r* = −0.430, *p* = 0.014) (Figures 6D,E, respectively) and positively correlated with S-VCAM-1 (*r* = 0.604, *p* < 0.001), S-IL-6 (*r* = 0.432, *p* = 0.002) and S-CRP (*r* = 0.324, *p* = 0.028) (Figures 6A–C respectively), as well as with usually recognized prognosis surrogates of ICU patients, such as APACHE II (*r* = 0.332, *p* = 0.026) and SAPS II (*r* = 0.302, *p* = 0.044) scores (Figures 6F,G, respectively).

U-Endocan was positively correlated with S-IL-6 (*r* = 0.398, *p* = 0.018), S-TNF-α (*r* = 0.373, *p* = 0.027) and U-protein/creatinine (*r* = 0.665, *p* < 0.001) (Figures 7A–C, respectively).

### Repeated measures multivariate analyses

The repeated measures multivariate analyses, considering all values during hospitalization and adjusted for age and gender, confirmed the positive relationship of S-Endocan with S-VCAM-1 previously detected in correlation analysis and evidenced a positive association with P-BNP, with higher values of these biomarkers being associated with higher S-Endocan values (Table 2). S-Endocan also had a borderline significant positive association with APACHE II score (Adjusted *β* = 0.377; 95% CI: −0.030–0.784, *p* = 0.069). The inverse relationships of S-Endocan with SBP or DBP were also confirmed in these multivariate analyses, with lower SBP and DBP values being associated with higher S-Endocan values (Table 2).

**TABLE 2:** Repeated measures multivariate models for S-Endocan in AHF and CS patients. (Adjusted *β*), 95% confidence intervals (95% CI) and *p* value estimated by repeated measures multivariate models with S-Endocan as the dependent variable and adjusted for age and gender.

S-Endocan (ng/ml)	Adjusted *β*	95% CI	*p* value
Model 1			
**S-VCAM-1** (ng/ml)	0.001	0.000 to 0.002	**0.005**
Model 2			
S-IL-6 (pg/ml)	0.000	-0.014 to 0.013	0.964
Model 3			
**P-BNP** (pg/ml)	0.002	0.000 to 0.04	**0.018**
Model 4			
P-hs-trop I (ng/L)	0.000	0.000 to 0.000	0.756
Model 5			
APACHE II score	0.377	−0.030 to 0.784	0.069
Model 6			
SAPS II score	0.133	−0.062 to 0.327	0.181
Model 7			
**SBP** (mmHg)	−0.193	−0.167 to - 0.038	**0.002**
Model 8
**DBP (mmHg)**	−0.144	−0.265 to −0.033	**0.011**

AHF, acute heart failure; APACHE II, acute physiology and chronic health evaluation; CS, cardiogenic shock; DBP, diastolic blood pressure; P-BNP, plasma B-type natriuretic peptide; P-hs-trop I, plasma high-sensitivity troponin I; SAPS II, simplified acute physiology score; SBP, systolic blood pressure; S-IL-6, serum interleukin 6; S-VCAM-1, serum vascular adhesion molecule 1. All the parameters that have significant *p* values (*p* < 0.05) should be written in bold (both the parameter and the respective *p* value).

### S-Endocan stratification by LVEF and mortality

When patients were stratified according to 2021 guidelines classification based on echocardiographic LVEF (McDonagh et al., 2021), admission S-Endocan values significantly increased in line with the degree of LVEF impairment (*p* for linear trend = 0.008) (Figure 8A). This was not observed for S-VCAM-1 values at admission (*p* for linear trend = 0.202) (Figure 8B).

Regarding the stratification of endocan values according to the mortality within 12 months, although non-survivors presented higher median values of S-Endocan at admission when compared to survivors (S-Endocan - Non-survivors: 7.9 (5.2; 13.5) ng/ml vs. Survivors: 6.2 (2.4; 9.8) ng/ml), these differences did not reach statistical significance (*p* = 0.158). Moreover, among CS patients, non-survivors presented higher median values of U-Endocan compared to survivors (U-Endocan—CS Non-survivors:10.3 (0.0; 100.2) pg/mg creatinine *vs*. CS Survivors: 0.0 (0.0; 25.6) pg/mg creatinine), although this was not statistically significant (*p* = 0.497).

### Performance of S-Endocan, U-Endocan, S-VCAM-1, P-BNP and prognostic scores as predictors of in-hospital mortality

APACHE II and SAPS II had similar and the highest AUC values for the prediction of in-hospital mortality, both being significant [AUC_APACHE II_: 0.790 (95% CI: 0.643–0.938), *p* = 0.002; AUC_SAPS II_: 0.807 (95% CI: 0.664–0.951), *p* = 0.001)] (Supplementary Table S2). When comparing confidence intervals, S-Endocan seemed to perform, at least, as well as P-BNP and S-VCAM-1 and also did not appear to be significantly different from APACHE II and SAPS II scores (Supplementary Table S2).

## Discussion

Our study highlights that inflammation-driven endothelitis might be a major pathophysiological mechanism across AHF spectrum, as evidenced by elevated concentrations of S-Endocan, S-VCAM-1, S-IL-6 and S-TNF-α both in AHF and CS group of patients. Of these parameters, S-Endocan seems the most promising biomarker since it not only raises with the hemodynamic severity of clinical presentation at admission but, most surprisingly, also increases with LVEF dysfunction. The positive correlation of S-Endocan with routine ICU prognostic scores and, particularly, its positive association with P-BNP in multivariate analyses suggest its relevance for inclusion in larger multimarker panels for AHF prognostication and eventually for future therapeutic targeting.

After many negative or neutral AHF trials, newer therapeutic targets are demanded once they are validated in pilot mechanistic studies where surrogate markers prove a solid and comprehensive inference for clinical benefit (Gheorghiade et al., 2005b). Endothelial dysfunction is prevalent and seems to be a predictor of adverse events in HF patients, also implicated in HF development and progression (Alem, 2019; Zuchi et al., 2020). Of note, therapies that have shown consistently to improve HF survival (e.g. angiotensin-converting enzyme inhibitors, spironolactone, beta blockers, etc) were demonstrated to simultaneously improve endothelial function (Marti et al., 2012). Therefore, a new interest in endothelial activation is re-emerging not only for risk stratification but also as a potential therapeutic target (De Keulenaer et al., 2017; Premer et al., 2019). The most validated theory considers that nitric oxide (NO) regulation of vascular tone contributes to the hemodynamic status in acute HF. Its imbalance with vasoconstrictors and reactive oxygen species determines increased vascular stiffness in pulmonary and systemic circulation and consequently ventricular workload and neurohormonal activation (Marti et al., 2012). Numerous clinical trials explored this therapeutically, either directly through drugs that modulate NO release, such as nebivolol (SENIORS trial) (Flather et al., 2005), or indirectly through drugs acting on cGMP-signalling axis, such as sildenafil (RELAX trial) (Redfield et al., 2013), riociguat (LEPHT study) (Bonderman et al., 2013) or vericiguat (SOCRATES trial) (Gheorghiade et al., 2015) and, ultimately, via the neprilysin inhibitor sacubitril that inhibits natriuretic peptide degradation causing amplification of the intracellular level of cGMP via natriuretic peptide receptor-A (McMurray et al., 2014). However, endothelial dysfunction extends beyond NO-mediated effects in the endothelium and its role in HF pathophysiology is more complex than previously anticipated. Endothelial dysfunction is now viewed as a common and important feature of all circulatory beds in HF, regardless of LVEF spectrum (Triposkiadis et al., 2019).

Although several non-invasive techniques have been developed for endothelial function testing, including flow-mediated vasodilation and finger plethysmography, these techniques are hardly implemented in the clinical daily practice. Due to these difficulties, some studies have quantified circulating endothelial biomarkers in HF patients, and, therefore, the measurement of many circulating endothelial biomarker candidates is becoming promising (Walczak et al., 2015). Circulating endothelial cells in the peripheral blood as well as E-selectin, von Willebrand factor and soluble thrombomodulin were shown to be significantly higher in patients with HF although they did not present significant differences between AHF and chronic HF and did not correlate with P-BNP (Chong et al., 2006a), ejection fraction or New York Heart Association (NYHA) class (Chong et al., 2006b). Also, VCAM-1 and ICAM-1 were shown to be associated with the development of new post-acute myocardial infarction HF symptoms (Lino et al., 2019) and increased subset-specific monocyte expression of their receptors were observed in AHF patients, with potential prognostic value of VCAM-1R (Wrigley et al., 2013) and sVCAM-1 after ST-elevation myocardial infarction (Hayek et al., 2021). But of all these, serum endocan has been one of the most qualified for the cardiovascular arena (Bessa et al., 2020).

In fact, in our work, most endothelial biomarkers, but not P-BNP, seem to differentiate AHF phenotype of presentation, since admission concentrations of S-ICAM-1, S-VCAM-1 and S-Endocan were significantly higher in CS patients. Among them, S-Endocan and S-VCAM-1 were also significantly increased in AHF patients, besides being linearly associated with the hemodynamic presentation of AHF. However, only S-Endocan increased in line with the degree of LVEF impairment evaluated by echocardiography. Thus, it may potentially be the first biomarker correlated with the severity of presentation of AHF phenotype and LVEF impairment, ultimately being anticipated as a better surrogate for prognostication in this entity if we consider hypoperfusion phenotype as the worst indicator for in-hospital mortality (Chioncel et al., 2019). This is corroborated by its positive association with P-BNP in repeated measures multivariate analysis adjusted for age and gender, as well as by its positive correlations with APACHE II and SAPS II. P-BNP (Metra et al., 2007) and LVEF (Solomon et al., 2007; O'Connor et al., 2008) are well established surrogates for prognosis in AHF and CS patients, although with some particularities and limitations (Chioncel et al., 2017a; Pang et al., 2019; Salah et al., 2019), whereas APACHE II and SAPS II are mostly validated for ICU patients in general. Accordingly, in our study, CS patients have longer ICU length of stay and tended to have a higher 1-year mortality, but there were no significant differences in in-hospital mortality between the AHF or CS groups. This might have resulted from a selection bias of AHF patients, which were only recruited from ICU, implicating selection of most severe patients, for example with need of non-invasive ventilation support. Moreover, the significantly older age of AHF patients and the higher prevalence of myocarditis (non-coronary etiologies) in CS group might also have contributed to these outcomes (Harjola et al., 2015). Noteworthy, we found that non-survivors had higher admission values of S-Endocan compared to survivors, although this did not reach statistical significance probably due to the aforementioned reasons and also to the exploratory nature of this small study, not designed to detect differences between survivors and non-survivors. We further assessed the performance of S-Endocan, U-Endocan, S-VCAM-1, P-BNP, APACHE II and SAPS II at admission to discriminate in-hospital mortality using ROC analysis. As already established, APACHE II and SAPS II are the best models for predicting mortality and this was confirmed again by their higher and significant AUC values. This was expected since they are a composite of clinical and analytical variables. However, when comparing confidence intervals, their performance did not appear to be significantly different from that of S-Endocan. This biomarker also seemed to perform, at least, as well as P-BNP and S-VCAM-1 in relation to in-hospital mortality, with the clinical advantage of being linearly associated with both hemodynamic presentation and ventricular dysfunction, which no analytical parameter demonstrated until now. These results are in line with previous studies, where for example, in ventilator-associated pneumonia, higher endocan concentrations were seen in non-survivors at day 1 and 7 (El Halim and Sayed, 2015) and also in patients with acute respiratory distress syndrome (ARDS) (Tang et al., 2014). In severe sepsis, a cut-off point was determined at days 1, 4, and 7, where higher endocan values were associated with poor prognosis (Hsiao et al., 2018) and with the need for mechanical ventilation (Mangat et al., 2017), thus representing a better biomarker than procalcitonin (Pauly et al., 2016; Zhao and Dong, 2017). Also remarkably, U-Endocan was even more evidently higher in CS non-survivors but again not statistically significant. Furthermore, its performance to discriminate in-hospital mortality did not appear to be significantly different from the other endothelial biomarkers tested.

Human endocan is synthetized by the vascular endothelium, namely by the pulmonary and kidney endothelial cells (Lassalle et al., 1996) but, unlike the other proteoglycans of the endothelial glycocalyx, it circulates freely in the bloodstream (Gaudet et al., 2017). The catabolism of endocan is not well known, resulting probably from proteolytic degradation and hepatic metabolism (De Freitas Caires et al., 2013; Nault et al., 2013). Urinary endocan is probably derived from kidney endothelial cells and in our work it was markedly higher in CS, suggesting locally increased renal endothelial dysfunction in these patients. Although it was positively correlated with proteinuria, which might indicate a relationship with glomerular lesion, we did not observe significant differences in renal function parameters between AHF and CS patients. Interestingly, urinary endocan was also positively correlated with inflammatory biomarkers such as IL-6 and TNF-α, reinforcing endocan association with inflammatory status. In contrast to our work, in populations with community acquired pneumonia, circulating endocan did not correlate with IL-6 or VCAM-1 at admission (Smart et al., 2018). This probably suggests that, in AHF, endocan is more timely linked to the inflammatory response and could potentially better reflect the complex multitude of pathophysiological pathways of AHF patients or even add incremental value to a multi-marker multi-point strategy of risk stratification (Lassus et al., 2013; Demissei et al., 2016).

We did not observe a reduction of endocan values or of other endothelial and inflammatory biomarkers during the first week of hospitalization in both patient groups. In other studies, namely on post-operative cardiac surgery patients, endocan was shown to peak very quickly at 6 h and slowly decline but it did not return to baseline at day 5 (Madhivathanan et al., 2016). Furthermore, higher endocan concentrations were observed in patients with the longest duration of norepinephrine support (Bougle et al., 2018) and could predict nosocomial pneumonia earlier and better than CRP (Perrotti et al., 2018). Endocan was also shown to increase during sepsis, worsening into multiple organ dysfunction syndrome (MODS), as well as to decrease when sepsis improves (Ioakeimidou et al., 2017). Severe sepsis with endocan concentrations remaining above 6.28 ng/ml at days 1, 4, and 7 was already demonstrated to be associated with poor prognosis (Hsiao et al., 2018). Of note, in our study, CS patients showed median S-Endocan values higher than 8 ng/ml throughout hospitalization. We hypothesize that in our patient groups, endocan kinetics could be more delayed than in the cases of sepsis already described, being specifically related to cardiovascular pathophysiology as evidenced by its linear association with ventricular dysfunction, but presenting similar prognostic implications.

Our results might indicate a perpetuation of endothelial dysfunction and inflammation in AHF and CS patients (Reina-Couto et al., 2021), reflecting the “non-resolving” course typical of this condition with high incidence of re-hospitalization and worse outcomes of AHF, as verified also in our population with median survival time of 13 months. This is in accordance with previous evidence in AHF patients showing that neurohormones and inflammatory biomarkers remain elevated 48h to 5 days after the acute event or even for longer periods (Milo et al., 2003; Gheorghiade et al., 2005a; Cotter et al., 2008). The persistence of these neurohormonal and inflammatory responses may be responsible for the high rehospitalization rates of this syndrome. So far, no therapeutic drug is well established as securely modifying prognosis in AHF (McDonagh et al., 2021) but current guidelines recommend decongestive therapy and eventually vasodilators for AHF patients who present with pulmonary edema or inotropes/vasopressors for CS patients (McDonagh et al., 2021). In fact, AHF or CS pharmacotherapy may be part of the problem. AHF pharmacological treatment may contribute to heightened neurohormonal activation (Goldsmith et al., 2018). Furthermore, the use of catecholamines as inotropes and/or vasopressors for the management of CS patients may further worsen cardiac and renal function (Amado et al., 2016; Tarvasmaki et al., 2016). Sympathetic activation is known to be associated with both inflammation and endothelial dysfunction and may underlie the unresolved inflammatory and endothelial responses in AHF and CS patients (Marvar and Harrison, 2012; Johansson et al., 2015; Ziegler et al., 2018). The therapeutic modulation of endocan has been explored in some studies as recently reviewed by our group (Bessa et al., 2020). However, as far as we know, no studies analysed the effects of mainstay pharmacotherapies for AHF (diuretics, vasodilators) or CS (inotropes, vasopressors) specifically on endocan. Regarding other drug treatments used in patients with cardiovascular disease, there is some evidence that RAAS inhibitors, statins and P2Y_12_ receptor antagonists can reduce endocan (Celik et al., 2015; Wei et al., 2017; Gao et al., 2018; Tuncez et al., 2019). However, in our study, only prior RAAS inhibitor treatment significantly decreased serum endocan and this was just observed for CS patients at admission. Treatment with statins or antiplatelet agents throughout hospitalization did not seem to affect serum or urinary endocan values in AHF or CS patients.

Endothelial dysfunction is poorly characterized in humans on mechanical circulatory support. In our study, we performed an extensive evaluation of endothelial and inflammatory biomarkers. Nevertheless, although the time frame selected in our study seemed adequate based on a previous study (Frerou et al., 2021), a more prolonged evaluation period might be required for VA-ECMO patients, ideally comparing pre- and post-cannulation. Indeed, during the first week of hospitalization, we could not observe any differences in the endothelial or inflammatory biomarkers in VA-ECMO patients compared to the conventional treatment group, with the exception of a tendential rise in S-CRP in the VA-ECMO group at days 5–8. These results are in contrast to a recent study describing that CS patients on VA-ECMO had higher values of plasma IL-6 and TNF-α within 24 h of VA-ECMO initiation compared to CS patients without VA-ECMO. Nevertheless, similarly, the authors could not find differences in these parameters between groups on day 4 after VA-ECMO initiation (Frerou et al., 2021). So far, from the scarce evidence found, there is still no consensus on the impact of ECMO on endothelial dysfunction and inflammation. Persistently high IL-6 concentrations in VA-ECMO patients were shown to be associated with poor prognosis (Risnes et al., 2008; Al-Fares et al., 2019), but in studies conducted in experimental models to investigate endothelial cell dysfunction associated with prolonged contact of blood components with synthetic surfaces, plasma from the ECMO experiments did not induce ICAM-1 expression in human umbilical vein endothelial cells during the 8 h of exposure (Graulich et al., 2000).

Major strengths of our study include an ICU population sample in “real-world conditions”, particularly CS and VA-ECMO patients, which are scarcely studied in the literature, as well as an extensive panel of endothelial and inflammatory biomarkers and the most commonly used clinical and echocardiographic prognostic indicators, including ICU APACHE II and SAPS II scores. Furthermore, our cohort appears to be representative of the mortality rate described in the literature for both AHF and CS (Chioncel et al., 2017b; Thiele et al., 2021). The small size of sample population and the unicentric character of this study are important limitations to point out that might limit the ability to generalize our results in terms of prognosis. Nevertheless, our results provide important clues of endocan usefulness as a prognostic marker in AHF and CS. We believe that endocan relation with mortality deserves to be explored in larger studies for its potential impact on disease management and prognosis. A further long-term prospective study should also be conducted after hospital discharge in patients presenting to ambulatory HF clinic since the results obtained throughout 1 week of hospitalization suggest that endothelitis might be a valuable therapeutic target in these patients.

In conclusion, admission concentrations of serum and urinary endocan significantly increase across AHF spectrum, but there is no reduction in the values of endocan or of other endothelial and inflammatory markers throughout hospitalization, suggesting a perpetuation of endothelial dysfunction and inflammation in these patients that could be related with the poor prognosis of this condition. Importantly, serum endocan appears to be a potential new biomarker of endothelitis and a putative therapeutic target in AHF and CS, being closely associated with LVEF impairment, BNP and prognostic scores.

## Data Availability

The raw data supporting the conclusion of this article will be made available on request by the authors, without undue reservation.

## Glossary

5-PL: five parameter logistic
AHF: acute heart failure
APACHE II: Acute Physiology And Chronic Health Evaluation II
ARDS: acute respiratory distress syndrome
AUC: area under curve;
CHUSJ: Centro Hospitalar Universitário São João
CKD-EPI: Chronic Kidney Disease Epidemiology Collaboration
CMT: conventional medical therapy
CS: cardiogenic shock
DBP: diastolic blood pressure
ECMO: extracorporeal membrane oxygenation
eGFR: estimated glomerular filtration rate
ELISA: enzyme-linked immunosorbent assay
ESM-1: endothelial cell-specific molecule-1
HF: heart failure
ICU: Intensive Care Unit
LV: left ventricular
LVEF: left ventricular ejection fraction
NO: nitric oxide
P-BNP: plasma B-type natriuretic peptide
P-hs-trop I: plasma high-sensitivity troponin I
RAAS: renin-angiotensin-aldosterone system
ROC: receiver operating characteristic
SAPS II: Simplified Acute Physiology Score II
SBP: systolic blood pressure
S-CRP: serum C-reactive protein
SEM: standard error of the mean
S-Endocan: serum endocan
S-E-Selectin: serum E-Selectin
S-ICAM-1: serum intercellular adhesion molecule one
S-IL-1β: serum interleukin one beta
S-IL-6: serum interleukin six
S-TNF-α: serum tumour necrosis factor alpha
S-Urea: serum urea
S-VCAM-1: serum vascular cell adhesion molecule one
sVCAM-1: soluble vascular cell adhesion molecule-1
U-Endocan: urinary endocan
VA-ECMO: veno-arterial extracorporeal membrane oxygenation
VCAM-1R: integrin-receptor to vascular cell adhesion molecule 1
