# Supplementary material for: Endothelitis profile in acute heart failure and cardiogenic shock patients: Endocan as a potential novel biomarker and putative therapeutic target
Source: Front Physiol. 2022 Aug 11;13:965611. doi: 10.3389/fphys.2022.965611 (PMC9407685; doi:10.3389/fphys.2022.965611)
Supplement: Supplementary file 1 [file Table1.pdf]

Supplementary Table S1. Impact of therapeutics on endothelial dysfunction markers in AHF and CS patients throughout hospitalization.

Previous therapeutics

RAAS inhibitors

	Admission			Days 3-4			Days 5-8		
	**Without previous RAAS inhibitors**	**With previous RAAS inhibitors**	***p* value**	**Without previous RAAS inhibitors**	**With previous RAAS inhibitors**	***p* value**	**Without previous RAAS inhibitors**	**With previous RAAS inhibitors**	***p* value**
**Acute Heart Failure**									
S-Endocan (ng/mL)	3.5 (2.1; 5.6)	3.5 (2.1; 7.4)	0.658	5.7 (2.9; 13.8)	3.0 (1.9; 6.5)	0.181	-	4.8 (1.0; 21.1)	-
U-Endocan (pg/mg creatinine)	0 (0; 0)	0 (0; 81)	0.458	0 (0; 26)	0 (0; 478)	>0.999	-	0 (0; 0)	-
S-ICAM-1 (ng/mL)	373 (239; 410)	326 (256; 469)	0.919	370 (270; 400)	357 (306; 511)	0.713	-	411 (312; 621)	-
S-VCAM-1 (ng/mL)	1493 (1167; 1902)	1218 (989; 2260)	0.865	1553 (1028; 2700)	2800 (1634; 3728)	0.264	-	3675 (1899; 5557)	-
S-E-Selectin (ng/mL)	28.2±4.5	34.9±2.6	0.206	28.1±4.7	38.6±4.5	0.147	-	35.3±6.5	-
**Cardiogenic Shock**									
S-Endocan (ng/mL)	**12.4 (8.5; 22.9)**	**8.1 (5.1; 12.2)**	**0.026**	13.4 (8.1; 22.0)	9.9 (4.0; 15.1)	0.424	8.5 (7.2; 13.1)	7.2 (3.2; 11.7)	0.360
U-Endocan (pg/mg creatinine)	24 (0; 57)	0 (0; 38)	0.524	15 (0; 284)	0 (0; 403)	>0.999	0 (0; 144)	5 (0; 363)	0.675
S-ICAM-1 (ng/mL)	478 (337; 626)	669 (420; 1016)	0.166	557 (348; 910)	474 (377; 975)	>0.999	497 (321; 629)	816 (401; 1113)	0.315
S-VCAM-1 (ng/mL)	3524 (2141; 5252)	1717 (1108; 2332)	0.058	3919 (2072; 4765)	2750 (1358; 3614)	0.408	3022 (2413; 3785)	2328 (1708; 3432)	0.315
S-E-Selectin (ng/mL)	30.6 (23.6; 47.2)	33.8 (20.8; 61.8)	0.563	33.0 (24.6; 42.9)	31.5 (23.4; 38.1)	0.536	35.1 (26.8; 38.3)	35.0 (22.7; 45.6)	0.829

Therapeutics during hospitalization

Statins

	Admission			Days 3-4			Days 5-8		
	**Without statins**	**With statins**	***p* value**	**Without statins**	**With statins**	***p* value**	**Without statins**	**With statins**	***p* value**
**Acute Heart Failure**									
S-Endocan (ng/mL)	3.3 (1.9; 5.7)	3.6 (2.2; 7.3)	0.538	6.4 (2.5; 22.0)	3.3 (1.9; 5.9)	0.446	-	4.8 (1.0; 21.1)	-
U-Endocan (pg/mg creatinine)	0 (0; 141)	0 (0; 0)	0.884	0 (0; 0)	0 (0; 34)	>0.999	-	0 (0; 0)	-
S-ICAM-1 (ng/mL)	267 (209; 420)	335 (278; 462)	0.403	331 (212; 378)	369 (323; 485)	0.317	-	411 (312; 621)	-
S-VCAM-1 (ng/mL)	1441 (986; 1904)	1343 (997; 2433)	0.971	1461 (701; 2144)	2800 (1485; 3745)	0.133	-	3675 (1899; 5557)	-
S-E-Selectin (ng/mL)	**22.9±3.4**	**36.0±2.4**	**0.015**	**21.7±3.8**	**39.0±3.7**	**0.025**	-	35.3±6.5	-
**Cardiogenic Shock**									

S-Endocan (ng/mL)	12.3 (8.6; 19.9)	8.9 (6.3; 14.5)	0.257	13.4 (9.2; 19.0)	9.9 (5.4; 15.6)	0.701	7.7 (5.8; 10.6)	8.8 (4.4; 13.1)	0.897
U-Endocan (pg/mg creatinine)	0 (0; 26)	10 (0; 100)	0.497	0 (0; 31)	0 (0; 453)	0.643	0 (0; 130)	18 (0; 363)	0.210
S-ICAM-1 (ng/mL)	483 (324; 737)	621 (355; 979)	0.410	432 (310; 655)	659 (382; 1087)	0.252	452 (313; 552)	751 (404; 1145)	0.122
S-VCAM-1 (ng/mL)	3812 (2346; 5855)	1729 (1100; 2880)	0.058	3919 (2148; 5343)	2750 (1409; 3895)	0.299	3269 (1613; 3838)	2738 (2127; 3067)	0.573
S-E-Selectin (ng/mL)	30.4 (22.4; 53.4)	34.5 (23.9; 54.8)	0.648	35.9 (26.7; 43.2)	31.5 (22.9; 35.5)	0.252	35.1 (26.5; 45.1)	35.0 (26.3; 38.9)	0.965

Aspirin

	**Admission**			**Days 3-4**			**Days 5-8**		
	**Without aspirin**	**With aspirin**	***p* value**	**Without aspirin**	**With aspirin**	***p* value**	**Without aspirin**	**With aspirin**	***p* value**
**Acute Heart Failure**									
S-Endocan (ng/mL)	3.3 (2.4; 4.9)	5.0 (2.0; 7.7)	0.600	3.3 (2.2; 8.6)	4.2 (2.0; 9.3)	>0.999	-	4.8 (1.0; 21.1)	-
U-Endocan (pg/mg creatinine)	0 (0; 0)	0 (0; 41)	0.737	0 (0; 0)	0 (0; 265)	0.500	-	0 (0; 0)	-
S-ICAM-1 (ng/mL)	267 (238; 343)	373 (295; 462)	0.072	**315 (241; 359)**	**417 (367; 559)**	**0.010**	-	411 (312; 621)	-
S-VCAM-1 (ng/mL)	1441 (1147; 2103)	1407 (889; 3026)	0.878	2071 (1246; 2815)	2409 (1237; 5180)	0.505	-	3675 (1899; 5557)	-
S-E-Selectin (ng/mL)	31.8±3.8	34.0±3.0	0.643	35.4±5.1	33.9±5.1	0.841	-	35.3±6.5	-
**Cardiogenic Shock**									
S-Endocan (ng/mL)	12.1 (8.5; 18.6)	9.3 (6.5; 16.1)	0.516	13.7 (6.4; 22.0)	9.9 (6.8; 15.1)	0.601	9.2 (5.2; 15.3)	8.4 (5.4; 11.7)	0.750
U-Endocan (pg/mg creatinine)	12 (0; 27)	0 (0; 57)	0.836	0 (0; 31)	0 (0; 453)	0.643	0 (0; 196)	9 (0; 357)	0.406
S-ICAM-1 (ng/mL)	487 (378; 866)	575 (352; 967)	0.829	432 (269; 883)	557 (385; 975)	0.377	452 (272; 704)	616 (401; 1063)	0.291
S-VCAM-1 (ng/mL)	**4101 (2814; 8568)**	**1717 (863; 2713)**	**0.007**	3919 (2776; 4765)	2750 (1459; 4176)	0.377	3269 (1835; 3785)	2738 (1768; 3463)	0.682
S-E-Selectin (ng/mL)	30.3 (22.0; 53.3)	32.9 (25.9; 54.2)	0.688	35.9 (22.3; 42.9)	31.8 (23.4; 38.1)	0.743	35.1 (24.6; 71.8)	34.8 (27.3; 38.4)	0.892

P2Y$_{12}$ receptor antagonists

	**Admission**			**Days 3-4**			**Days 5-8**		
	**Without P2Y$_{12}$ receptor antagonists**	**With P2Y$_{12}$ receptor antagonists**	***p* value**	**Without P2Y$_{12}$ receptor antagonists**	**With P2Y$_{12}$ receptor antagonists**	***p* value**	**Without P2Y$_{12}$ receptor antagonists**	**With P2Y$_{12}$ receptor antagonists**	***p* value**
**Acute Heart Failure**									
S-Endocan (ng/mL)	3.3 (2.4; 6.7)	4.4 (2.1; 7.6)	>0.999	3.4 (2.8; 9.7)	3.1 (1.9; 8.3)	0.536	15.7 (1.0; 30.3)	4.8 (1.0; 11.9)	>0.999
U-Endocan (pg/mg creatinine)	0 (0; 0)	0 (0; 41)	0.864	0 (0; 0)	0 (0; 34)	>0.999	0 (0; 0)	0 (0; 0)	-
S-ICAM-1 (ng/mL)	**267 (245; 333)**	**398 (341; 462)**	**0.004**	**299 (234; 362)**	**383 (357; 535)**	**0.016**	305 (283; 327)	543 (365; 680)	0.267
S-VCAM-1 (ng/mL)	1218 (989; 2103)	1508 (1018; 3026)	0.446	2281 (1731; 2829)	1816 (1138; 4772)	0.918	1541 (823; 2258)	4853 (3116; 6845)	0.133

S-E-Selectin (ng/mL)	29.9±2.9	37.3±3.4	0.115	33.5±5.5	35.6±4.8	0.770	26.4±5.4	39.7±9.0	0.391
**Cardiogenic Shock**									
S-Endocan (ng/mL)	12.4 (8.5; 18.7)	8.3 (5.6; 13.5)	0.148	14.4 (10.3; 23.4)	8.1 (4.7; 12.8)	0.088	9.0 (5.4; 13.1)	8.4 (3.8; 11.3)	0.697
U-Endocan (pg/mg creatinine)	10 (0; 25)	0 (0; 144)	0.603	0 (0; 23)	184 (0; 495)	0.429	0 (0; 72)	26 (0; 369)	0.275
S-ICAM-1 (ng/mL)	589 (384; 839)	485 (352; 1004)	0.976	495 (314; 696)	566 (385; 1144)	0.328	497 (319; 851)	616 (401; 1230)	0.360
S-VCAM-1 (ng/mL)	**3698 (2497; 6005)**	**1435 (770; 2204)**	**<0.001**	4048 (2914; 5054)	2215 (1384; 3579)	0.083	2960 (2247; 3785)	2593 (1494; 3463)	0.515
S-E-Selectin (ng/mL)	36.3 (23.6; 54.1)	30.7 (20.0; 46.6)	0.522	37.0 (28.2; 43.0)	27.6 (22.7; 32.7)	0.161	38.0 (31.4; 41.8)	29.5 (22.4; 36.8)	0.203

RAAS, renin-angiotensin-aldosterone system; S-Endocan, serum endocan; S-ICAM-1, serum intercellular adhesion molecule 1; S-VCAM-1, serum vascular cell adhesion molecule 1; U-Endocan, urinary endocan. Results are expressed as mean±SEM or as median (25th percentile; 75th percentile) for data with normal or non-normal distribution, respectively.
