# Supplementary material for: Endothelitis profile in acute heart failure and cardiogenic shock patients: Endocan as a potential novel biomarker and putative therapeutic target
Source: Front Physiol. 2022 Aug 11;13:965611. doi: 10.3389/fphys.2022.965611 (PMC9407685; doi:10.3389/fphys.2022.965611)
Supplement: Supplementary file 2 [file Table2.pdf]

Supplementary Table S2. Performance of S-Endocan, U-Endocan, S-VCAM-1, P-BNP and prognostic scores as predictors of in-hospital mortality

	**AUC**	**95% CI**	***p* value**
S-Endocan (ng/mL)	0.626	0.466-0.787	0.143
U-Endocan (pg/mg creatinine)	0.597	0.402-0.793	0.330
S-VCAM-1 (ng/mL)	0.581	0.418-0.743	0.348
P-BNP (pg/mL)	0.614	0.417-0.811	0.237
**APACHE II**	0.790	0.643-0.938	**0.002**
**SAPS II**	0.807	0.664-0.951	**0.001**

APACHE II, Acute Physiology And Chronic Health Evaluation; AUC, Area under Curve; P-BNP, plasma B-type natriuretic peptide; S-Endocan, serum endocan; SAPS II, SAPS, Simplified Acute Physiology Score; S-VCAM-1, serum vascular cell adhesion molecule 1; U-Endocan, urinary endocan.
