# Supplementary figures and images for: Endothelitis profile in acute heart failure and cardiogenic shock patients: Endocan as a potential novel biomarker and putative therapeutic target
Source: Front Physiol. 2022 Aug 11;13:965611. doi: 10.3389/fphys.2022.965611 (PMC9407685; doi:10.3389/fphys.2022.965611)

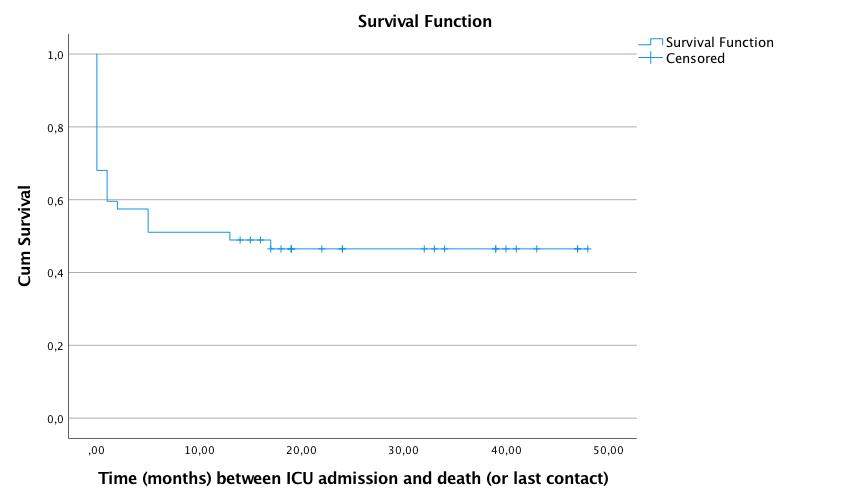

Supplement: Supplementary file 3 [file Image1.jpg]
